# ExSPIN: Explicit Feedback-Based Self-Play Fine-Tuning for Text-to-SQL Parsing

**DOI:** 10.3390/e27030235

**Published:** 2025-02-25

**Authors:** Liang Yan, Jinhang Su, Chuanyi Liu, Shaoming Duan, Yuhao Zhang, Jianhang Li, Peiyi Han, Ye Liu

**Affiliations:** 1 School of Computer Science and Technology, Harbin Institute of Technology, Shenzhen 518055, China; yanlianginspurhit@gmail.com (L.Y.); 24s151151@stu.hit.edu.cn (J.S.); 200110416@stu.hit.edu.cn (Y.Z.); 220110828@stu.hit.edu.cn (J.L.); 2 Inspur Cloud Information Technology Co., Ltd., Jinan 250101, China; 3 Pengcheng Laboratory, Shenzhen 518000, China; 4 Key Laboratory of Cyberspace and Data Security, Ministry of Emergency Management, Beijing 100010, China; 5 Guangdong Power Grid Co., Ltd., Guangzhou 510000, China; sz170604510755@163.com

**Keywords:** large language model, text-to-SQL, self-play fine-tuning

## Abstract

Recently, self-play fine-tuning (SPIN) has garnered widespread attention as it enables large language models (LLMs) to iteratively enhance their capabilities through simulated interactions with themselves, transforming a weak LLM into a strong one. However, applying SPIN to fine-tune text-to-SQL models presents substantial challenges. Notably, existing frameworks lack clear signal feedback during the training process and fail to adequately capture the implicit schema-linking characteristics between natural language questions and databases. To address these issues, we propose a novel self-play fine-tuning method for text-to-SQL models, termed ExSPIN, which incorporates explicit feedback. Specifically, during fine-tuning, the SQL query execution results predicted by the LLM are fed back into the model’s parameter update process. This feedback allows both the main player and the opponent to more accurately distinguish between negative and positive samples, thereby improving the fine-tuning outcomes. Additionally, we employ in-context learning techniques to provide explicit schema hints, enabling the LLM to better understand the schema-linking between the database and natural language queries during the self-play process. Evaluations on two real-world datasets show that our method significantly outperforms the state-of-the-art approaches.

## 1. Introduction

Text-to-SQL parsing [1] involves translating user queries in natural language into executable SQL queries that can be run on databases. Text-to-SQL allows users, even those without knowledge of SQL or database techniques, to interact with databases using natural language, and it has attracted increasing attention from both the database and natural language processing communities. With the advent of large language models (LLMs), numerous methods [2,3,4] have demonstrated that LLMs can significantly improve the accuracy of text-to-SQL parsers. However, all of these methods are based on closed-source models, such as GPT-4 [5], raising concerns about data leakage in private scenarios.

Recently, the emergence of a large number of open-source LLMs [6,7] has attracted widespread attention because these models show capabilities comparable to those of closed-source models in a wide range of natural language processing (NLP) tasks. Recent studies [8,9,10] have demonstrated that open-source LLMs with fewer parameters, when fine-tuned on human-annotated data, can achieve SQL generation accuracy comparable to large closed-source LLMs. This makes fine-tuning open-source LLMs a viable solution for private scenarios. While effective, these methods are costly due to their reliance on manually annotated data. To address this, researchers are exploring alternative fine-tuning approaches that reduce the human dependency. This motivates us to study the fine-tuning of LLMs without the need for extra human-annotated data to convert weak models to strong models in text-to-SQL tasks.

In response to the challenge of data scarcity, numerous studies [11,12,13,14] have explored self-play mechanisms to iteratively improve models by engaging in “competitive interactions” with their own generated instances. This approach has been proven successful in systems like AlphaGo Zero [11] and AlphaZero [12]. To enhance the performance of weak LLMs, SPIN [14] proposes improving models by allowing them to play against themselves, without requiring direct supervision. This approach autonomously generates data, progressively enhancing the model’s capabilities while maximizing the utility of corrected labeled examples in supervised fine-tuning (SFT).

**Figure 1:**
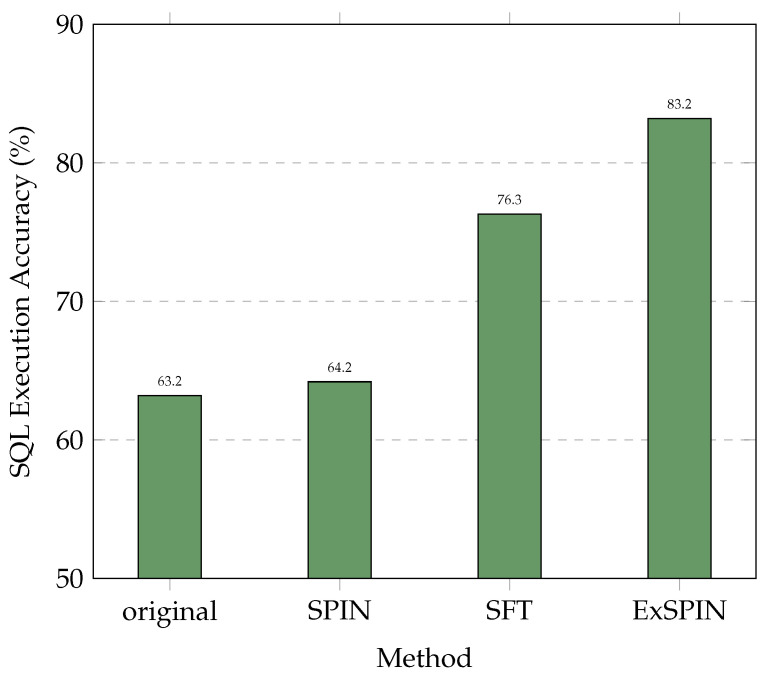
Comparison results on the SPIDER [16] dataset. The original represents DeepSeek-Coder v1 6.7B [6], while SFT, SPIN, and ExSPIN denote the models fine-tuned on the training set using SFT, SPIN, and ExSPIN, respectively.

Although SPIN enhances the data diversity, its application to text-to-SQL tasks remains unexplored. As shown in Figure 1, directly applying SPIN to text-to-SQL models leads to a significant performance drop. In some cases, the performance falls below that of the original model and the model fine-tuned via SFT. This performance degradation primarily arises from two key issues. First, in the text-to-SQL task, SQL queries have well-defined execution results, but SPIN does not leverage this clear feedback. Instead, it uses the semantic similarity between SQL queries to distinguish between positive and negative samples. However, in practice, two semantically similar SQL queries may yield different execution results, making it challenging for the model to generate correct SQL queries. Second, the data synthesized by SPIN neglect the implicit link between natural language questions and database schemas [15], preventing the model from learning the true relationships within the data.

To address these challenges, we propose a novel framework, ExSPIN, which incorporates explicit information—specifically, SQL execution feedback and schema information—into self-play fine-tuning. First, before each round of iterative training, we introduce an explicit schema integration method that incorporates schema information relevant to natural language questions. This method constructs display prompts to bridge the mismatch between natural language queries and database schemas in the large model. In each training round, we also propose an execution feedback fine-tuning method. This method changes the opponent model’s strategy for distinguishing between positive and negative samples. Instead of relying on semantic similarity, it focuses on the execution results of SQL queries predicted by the opponent model. This explicit feedback improves the opponent model’s ability to evaluate synthetic data’s quality, enhancing the primary model’s performance. We evaluate ExSPIN on two real-world datasets: SPIDER and BIRD. The experimental results show that LLMs fine-tuned with ExSPIN outperform state-of-the-art (SOTA) methods.

Our main contributions are as follows.

1. We propose a SPIN framework that explicitly integrates SQL execution feedback and schema information into the training process, enhancing the model’s understanding of the data and, consequently, improving its accuracy.
2. We present a method for explicit schema integration in self-play training, which accurately extracts target schema information and mitigates the influence of noise.
3. We introduce an execution feedback fine-tuning method that incorporates SQL execution results into the model’s parameter update process, thereby improving the performance of self-play fine-tuning.
4. We conduct experiments on two real-world datasets, and the results demonstrate that ExSPIN effectively enhances model performance, surpassing the state-of-the-art (SOTA) methods.

## 2. Related Work

### 2.1. Self-Play Fine-Tuning

Self-play, a technique where agents interact with copies or past versions of themselves, has emerged as a powerful method in reinforcement learning (RL), particularly in multi-agent settings. This approach has been instrumental in solving complex problems in games such as Go, chess, poker, and video games, where agents have developed strategies that surpass human expertise [17]. Self-play addresses several inherent challenges in multi-agent reinforcement learning (MARL), such as non-stationarity and coordination, by allowing agents to learn in a more stable and manageable environment.

Traditional self-play operates within the framework of Markov decision processes (MDPs) [18] and extends to multi-agent settings through Markov games, also known as stochastic games. In these settings, agents interact with an environment by taking actions, transitioning between states, and receiving rewards. The goal of RL algorithms is to derive an optimal policy that maximizes the expected accumulated reward over time. Deep RL [19], which employs deep neural networks as function approximators, has further enhanced the ability to handle high-dimensional state spaces, leading to breakthroughs in various complex tasks. In MARL, the interdependence of agents’ actions introduces significant challenges, as the environment appears non-stationary to each agent [20]. Self-play offers a solution by allowing agents to interact with copies or past versions of themselves, thereby reducing the complexity of the learning process. This method has been particularly effective in competitive scenarios, where agents must learn to coordinate and compete simultaneously.

Self-play algorithms can be broadly categorized into four groups: traditional self-play algorithms, the Policy-Space Response Oracles (PSRO) series [21], ongoing training-based algorithms, and regret minimization-based algorithms. Self-play has been successfully applied in various domains, including board games, card games, and video games. In board games like Go and Chess, self-play has led to the development of agents that surpass human performance. For example, AlphaGo [11] and its successors (AlphaGo Zero, AlphaZero, and MuZero) have revolutionized the field by leveraging self-play to achieve superhuman performance without relying on expert data. In card games like Texas Hold’em and DouDiZhu [22], self-play has been used to develop agents that can compete with and defeat professional players. Techniques such as abstraction and continual re-solving have been employed to handle the complexity of these games. Similarly, in video games like StarCraft II and Dota 2, self-play has enabled agents to master complex real-time strategies, often outperforming human players.

Recently, self-play has found applications in enhancing large language models (LLMs), as exemplified by the Self-Play Fine-Tuning (SPIN) framework [14]. SPIN offers a novel approach to converting weak LLMs into strong ones by leveraging iterative self-improvement. Unlike traditional methods that heavily rely on human-annotated datasets or additional preference data for fine-tuning, SPIN eliminates the need for external supervision after the initial supervised fine-tuning (SFT) phase. This distinguishes it from prevalent techniques such as reinforcement learning from human feedback (RLHF) [23] and direct preference optimization (DPO) [24], both of which require costly human feedback or advanced model-guided preferences.

In SPIN, a model iteratively generates its synthetic training data, allowing the current iteration to refine its policy by distinguishing between self-generated and human-annotated responses. This methodology aligns with the broader objective of leveraging synthetic data in fine-tuning tasks, as seen in recent research exploring data augmentation strategies for LLMs. Theoretically, SPIN ensures convergence to a global optimum where the model’s policy aligns with the target data distribution. Empirically, the method has shown significant improvements across various benchmarks, such as HuggingFace’s Open LLM Leaderboard and MT-Bench, surpassing models fine-tuned with additional preference datasets. Furthermore, SPIN exhibits parallels with adversarial training frameworks like generative adversarial networks (GANs) [25], where the generator (model from the prior iteration) and discriminator (current model) engage in a competitive dynamic to enhance model robustness.

### 2.2. In-Context Learning for Text-to-SQL Parsing

With the rapid progress in natural language processing, in-context learning (ICL) has emerged as a paradigm enabling zero-shot or few-shot learning across various tasks by providing a small number of examples without additional training [26]. This capability is particularly valuable in applications requiring adaptability to novel data and domains. The text-to-SQL task, which involves converting natural language queries into structured SQL queries, presents significant challenges due to the diversity of natural language and the rigid syntax of SQL. In this domain, ICL has demonstrated strong adaptability.

Traditional approaches to the text-to-SQL task often rely on supervised learning and require large-scale annotated datasets for model training. In contrast, ICL incorporates contextual examples directly into the input, allowing pre-trained language models to infer SQL queries with minimal examples. The GPT-3 [27] model highlighted the potential of ICL, showcasing how task examples could enable the model to perform tasks without additional fine-tuning. Subsequent research has shown that the careful selection of the number, order, and content of contextual examples can significantly improve model performance in specific domains.

In the text-to-SQL task, researchers have explored various ICL strategies. For example, by providing structured natural language–SQL pairs as examples, models can better interpret the intent of input queries and generate corresponding SQL statements [2]. Even in scenarios lacking abundant annotated data, ICL leverages the intrinsic knowledge of large pre-trained language models, producing SQL queries with strong syntactic correctness and query accuracy.

### 2.3. SFT-Based Text-to-SQL

In recent years, supervised fine-tuning (SFT) methods have made significant progress in the text-to-SQL task, enhancing the accuracy and robustness of SQL generation by fine-tuning models with annotated data. MEDT5SQL [28] is a text-to-SQL method designed for the healthcare domain. Through domain-adaptive pre-training and multi-task learning, it optimizes a T5 model and significantly improves its performance on healthcare databases. SQL-PaLM [29] leverages the closed-source large-scale pre-trained language model PaLM. It optimizes the model’s ability to generate SQL queries through task-specific fine-tuning strategies, such as SQL syntax enhancement, data augmentation, and a query complexity-aware loss function. RASAT + PICARD [30] combines the relationally aware self-attention mechanism (RASAT) with a post-processing technique, PICARD. The former enhances the model’s understanding by capturing relationships in the database schema, while the latter ensures that the generated SQL queries conform to syntax and semantic rules through constrained decoding. RESDSQL-3B + NatSQL [31] employs a large-scale pre-trained model with 3 billion parameters, combined with NatSQL (an intermediate representation from natural language to SQL). It generates more natural and accurate SQL queries through a multi-stage fine-tuning strategy. Finally, TREQS [32] introduces a template-based text-to-SQL method that leverages predefined SQL templates and template selection strategies to quickly generate syntactically correct queries, reducing the likelihood of incorrect queries. In contrast to the aforementioned methods, our approach innovatively introduces self-play into the text-to-SQL task. By filtering out erroneous SQL queries generated by an adversarial model, we guide the main model to correct its own errors through interaction with the adversarial model. Compared to other fine-tuning methods, our approach achieves intrinsic self-improvement without requiring additional manually labeled data and automatically synthesizes data for fine-tuning.

## 3. Problem Setting and Preliminaries

In the SPIN framework, the primary player model aims to distinguish between responses generated by the LLM and those generated by humans, while the adversary model strives to generate responses that are indistinguishable from human responses. The core of SPIN lies in its self-adversarial mechanism, where both the primary player and the adversary are instances of the same LLM but from different iterations. Specifically, the adversary is the version of the LLM from the previous iteration, while the primary player is the LLM being trained in the current iteration. In iteration t+1, the adversary LLM from the previous iteration, denoted as $p_{\theta_{t}}$, generates a response $y^{\prime }$ from the prompt *x* according to $p_{\theta_{t}}\left(\cdot |x\right)$. Therefore, the optimization objective of the SPIN in iteration t+1 is

(1) $$f_{t+1}=\underset{f\in F_{t}}{\mathrm{argmax}}E\left[f\left(x,y\right)-f\left(x,y^{\prime }\right)\right]$$

The objective of the primary player $f_{t+1}$ is to maximize the expected difference between the scores assigned to human responses *y* and adversary responses $y^{\prime }$. SPIN defines the closed-form solution for this optimization objective $F_{t}$ as follows:

(2) $$F_{t}=\left\{\lambda \cdot \log\frac{p_{\theta }\left(y|x\right)}{p_{\theta_{t}}\left(y|x\right)}|\theta \in \Theta \right\}$$

where Θ is the parameter space of the considered LLM. Given $F_{t}$ in Equation (2), we obtain the parameterized function for $f_{t+1}$ in SPIN:

(3) $$f_{t+1}\left(x,y\right)=\lambda \cdot \log\frac{p_{\theta_{t+1}}\left(y|x\right)}{p_{\theta_{t}}\left(y|x\right)}.$$

Here, $p_{\theta_{t+1}}\left(y|x\right)$ and $p_{\theta_{t}}\left(y|x\right)$ represent the similarity between the responses generated by the model and the human responses *y*. The capability of the main model is evaluated by calculating the logarithmic ratio of these two similarities. If the main model can generate responses that align more closely with the human responses compared to the adversary model, the value of $f_{t+1}\left(x,y\right)$ will be higher.

Substituting this parameterized function into the optimization objective, we derive an end-to-end training objective and the update rule for $\theta_{t+1}$:

(4) $$L_{\mathrm{SPIN}}=E\left[\ell \left(\lambda \log\frac{p_{\theta }\left(y|x\right)}{p_{\theta_{t}}\left(y|x\right)}-\lambda \log\frac{p_{\theta }\left(y^{\prime }|x\right)}{p_{\theta_{t}}\left(y^{\prime }|x\right)}\right)\right]$$

where the expectation is computed over the distribution:

(5) $$x∼q\left(\cdot \right),y∼p_{\mathrm{data}}\left(\cdot |x\right),y^{\prime }∼p_{\theta_{t}}\left(\cdot |x\right).$$

Formula (4) represents the final training objective of SPIN. It uses the loss function *ℓ* to measure the difference between the output of the primary player $f_{t+1}$ and the target. Specifically, it calculates the difference in the log-probability ratios between the preferences of the two models for human responses *y* and adversary responses $y^{\prime }$. By optimizing this difference through *ℓ*, the objective function guides the primary model to generate responses that are more similar to human responses and reduces the probability of generating responses similar to those from the adversary model.

However, in the text-to-SQL task, the SPIN framework primarily suffers from the following two issues. First, the SPIN adversarial model ignores the implicit link between natural language questions and database schemas. Specifically, in the SPIN optimization function, the model only increases the reward for the correct SQL $\log p_{\theta }\left(y|x\right)$ and imposes a penalty on the responses generated by the adversary model $p_{\theta_{t}}\left(\cdot |x\right)$: $\log\frac{1}{p_{\theta }\left(y^{\prime }|x\right)}$, to enhance the primary model. However, in the text-to-SQL task, the accuracy of SQL generation often depends on the model’s ability to understand the relationship between the question and the database. The traditional SPIN framework can only implicitly learn the correspondence between the question and the database by favoring correct SQL queries, making it challenging for the model to deepen its understanding of schema-linking features. As a result, this leads to suboptimal performance in SQL generation tasks.

Second, SPIN overemphasizes exact SQL matching. Unlike text generation tasks, which often focus on human preferences, the text-to-SQL task typically requires the model to generate SQL statements that align with the query intent, rather than producing SQL statements that are identical to human-annotated ones. Achieving exact matches with annotated SQL is not only difficult but also undermines the model’s robustness. Instead, we prefer the model to generate diverse SQL statements that still retrieve the correct data, which would significantly enhance its generalization ability. However, in the traditional SPIN framework, the model is trained to generate SQL statements identical to the annotated ones, while dismissing its own generated statements that are correct. Specifically, for the correctly executable SQL statements $y_{right}^{\prime }$ generated by the adversary model, the update for the primary player model becomes

(6) $$L_{\mathrm{SPIN}}=E\left[\ell \left(\lambda \log\frac{p_{\theta }\left(y|x\right)}{p_{\theta_{t}}\left(y|x\right)}-\lambda \log\frac{p_{\theta }\left(y_{right}^{\prime }|x\right)}{p_{\theta_{t}}\left(y_{right}^{\prime }|x\right)}\right)\right]$$

This optimization function penalizes $p_{\theta_{t}}\left(y_{right}^{\prime }|x\right)$, causing the model to modify its output. However, when the output is correct, this penalty harms the model’s robustness, leading to a decline in the accuracy of the generated SQL.

## 4. ExSPIN

**Figure 2:**
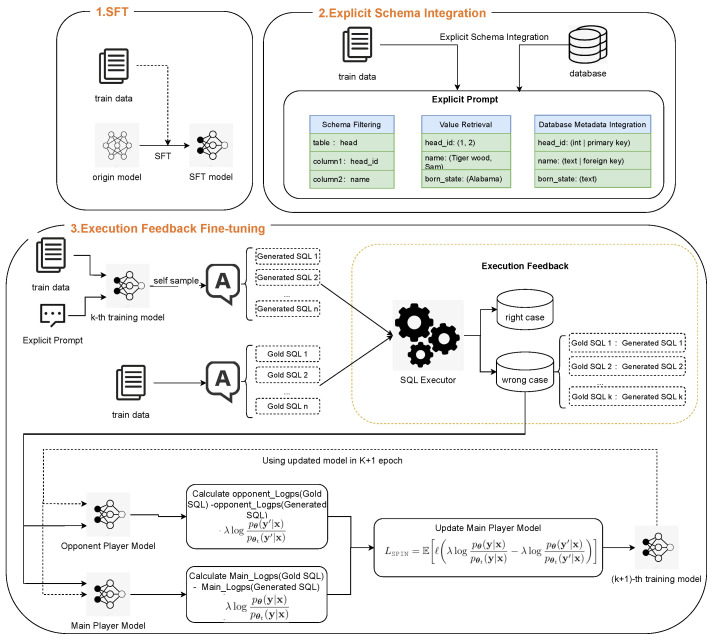
The workflow of ExSPIN.

To address the challenges faced by traditional SPIN methods in text-to-SQL parsing tasks, we propose an explicit feedback-based self-play fine-tuning (ExSPIN) framework. The framework consists of three stages: supervised fine-tuning (SFT), explicit schema integration, and execution feedback self-play fine-tuning. As shown in Figure 2, we first fine-tune a text-to-SQL model using supervised fine-tuning (SFT) techniques, enabling it to generate SQL queries based on natural language instructions. Next, we apply explicit schema integration to construct training prompts from natural language questions, injecting relevant database schema information to capture the intent of the query. Finally, execution feedback fine-tuning incorporates the execution results of the SQL queries generated by the adversarial model into the parameter update process. The following sections provide a detailed description of each stage of the process.

### 4.1. Supervised Fine-Tuning

In this subsection, we introduce the first step of ExSPIN. The goal of SFT is to fine-tune an LLM with preliminary SQL generation capabilities according to the natural language question. During the fine-tuning process, the model maps natural language queries to corresponding SQL statements through supervised learning. Given $\left(x_{i},y_{i}\right)\in D_{train}$, we apply the standard supervised fine-tuning (SFT) objective on the base model *P* with parameters θ:

(7) $$L\left(\theta \right)=\sum_{\left(x_{i},y_{i}\right)\in D_{\mathrm{train}}}\log P_{\theta }\left(y_{i}|x_{i}\right)$$

where $x_{i}$ denotes the *i*-th input, consisting of a concatenated instruction and user query.

### 4.2. Explicit Schema Integration

**Figure 3:**
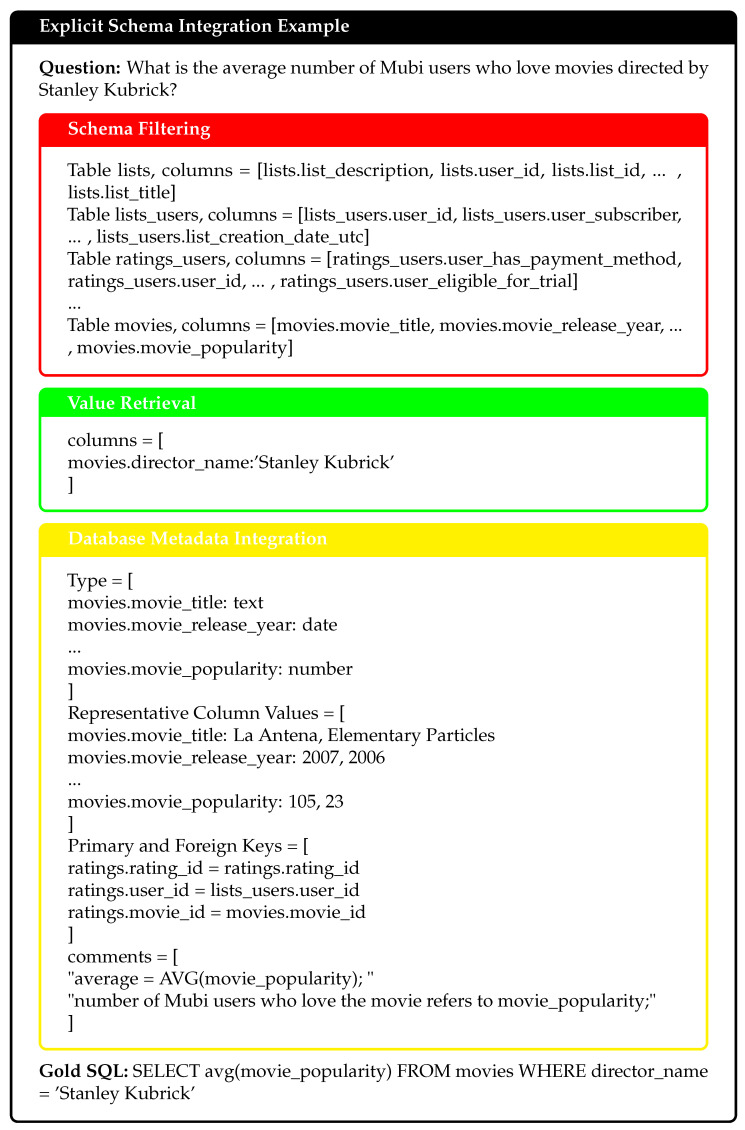
Explicit schema integration example.

Explicit schema integration aims to embed schema-linking features between natural language questions and database schemas into the model’s input during training. These well-structured prompts help the model to better understand the relationships between database elements. This significantly improves its ability to generate accurate SQL queries. Our method focuses on three key components: schema filtering, value retrieval, and database metadata integration. Figure 3 provides a practical example output of explicit schema integration. Given the query “What is the average number of Mubi users who love movies directed by Stanley Kubrick?”, through schema filtering, relevant tables and columns are identified; value retrieval extracts the specific query-related value “Stanley Kubrick”; and database metadata integration incorporates column types, representative values, and key relationships.

**Schema Filtering.** To ensure that the database schemas are closely aligned with the input query, we retrieve the most relevant tables and columns to generate the target SQL query. To achieve this, we design a schema filter *f* that evaluates the relevance of database tables *T* and columns *C* with respect to a given query *Q*. The filter assigns a relevance score as follows:

(8) $$score_{T_{i},C_{ij}}=f\left(Q,T_{i},C_{ij}\right),\;i=1,2,\ldots ,t$$

We include the top $k_{1}$ tables and $k_{2}$ columns with the highest relevance scores in the training prompt. In cases where fewer than $k_{1}$ tables are deemed relevant, we supplement the prompt with randomly selected tables from the database. This strategy minimizes token usage while preserving the essential schema information needed to generate accurate SQL queries.

**Value Retrieval.** Incorporating query-specific values from the database into the prompt is crucial for accurate SQL generation. For example, given the query “How many people live in Shanghai?”, the database column “city.name” containing the value “Shanghai” should result in the condition “city.name = ‘Shanghai”’ being included in the prompt. However, directly retrieving values from a large database can be computationally expensive. To address this challenge, we employ a two-stage approach. First, we construct a BM25 index [33] to perform a coarse search, narrowing down the potential matches. Then, we apply the longest common substring (LCS) algorithm to identify the specific values corresponding to the query. This coarse-to-fine strategy effectively reduces the computational overhead while maintaining high retrieval accuracy.

**Database Metadata Integration.** To further enrich schema-linking, we incorporate key metadata elements into the prompt as follows.

- Primary and Foreign Keys: Incorporating primary and foreign key information can help the model to deduce the appropriate join path. We extract this information to establish relationships between tables and guide the model in constructing accurate JOIN operations.
- Representative Column Values: By including representative values for each column (e.g., human.gender = F|M), we enhance the model’s understanding of the column content and format.
- Column Data Types: The types of column data dictate the validation rules and permissible operations, ensuring accurate SQL query formulation. For instance, numeric columns require casting when performing arithmetic operations if stored as strings.
- Comments: Database comments help to clarify ambiguities in schema elements and enable the LLM to perform precise schema-linking. For example, by adding the comment “time consumed per training round”, the model can understand the true meaning of the column name “tcpr”.

Algorithm 1 summarizes the procedures of our explicit schema integration process. By building these high-quality prompts, we significantly improve the model’s capacity to generate precise SQL queries during SPIN.    
**Algorithm 1:** Explicit Schema Integration
     **Input**: User question *Q*, schema item classifier model *f*, database schema $D_{\mathrm{schema}}$, database                 metadata $D_{\mathrm{meta}}$, database index *I*, maximum table and column numbers $k_{1}$, $k_{2}$
**Output**: Database prompt $Prompt_{\mathrm{db}}$
**        // Schema Filtering**$score_{T_{i},C_{ij}}=f\left(Q,T_{i},C_{ij}\right)$,    i=1,2,…,t;$P_{\mathrm{schema}}=\mathrm{SelectTableColumn}\left(score_{T_{i},C_{ij}},D_{\mathrm{schema}},k_{1},k_{2}\right)$;**        // Value Retrieval**$V_{\mathrm{coarse}-\mathrm{grained}}=\mathrm{Search}-\mathrm{by}-\mathrm{Index}\left(Q,I\right)$;$V_{\mathrm{fine}-\mathrm{grained}}=\mathrm{LCS}\left(Q,V_{\mathrm{coarse}-\mathrm{grained}}\right)$;**        // Metadata Integration and Prompt Concatenation**$S_{\mathrm{meta}}=\mathrm{Serialize}\left(D_{\mathrm{meta}}\right)$;$S_{\mathrm{schema}}=\mathrm{Serialize}\left(P_{\mathrm{schema}}\right)$;$S_{\mathrm{value}}=\mathrm{Serialize}\left(V_{\mathrm{fine}-\mathrm{grained}}\right)$;$Prompt_{\mathrm{db}}=\mathrm{ConcatSequence}\left(S_{\mathrm{schema}},S_{\mathrm{meta}},S_{\mathrm{value}}\right)$**return** $Prompt_{\mathrm{db}}$;


### 4.3. Execution Feedback Fine-Tuning

After explicit schema integration, we obtain the training input prompt of the SPIN. In the t+1-th iteration, we concatenate the database prompt text *P* with the query input *x*, denoted as $x^{\prime }$, which is represented as

(9) $$x^{\prime }=\left[Q;x\right]$$

With this enhanced input, we train the main model and the adversary model. In the SPIN training process, the goal of the main model is to distinguish between the outputs of the adversary model and the original dataset outputs. After enhancing the input with the database prompt text, the main model $f_{t+1}$ maximizes the expected gap between the target data distribution $p_{\mathrm{data}}$ and the adversary model distribution $p_{\theta_{t}}$:

(10) $$f_{t+1}=\arg\max_{f\in F_{t}}E\left[f\left(x^{\prime },y\right)-f\left(x^{\prime },y^{\prime }\right)\right]$$

where the expectation is computed over the following distributions: $x^{\prime }∼q\left(\cdot \right)$, $y∼p_{\mathrm{data}}\left(\cdot |x^{\prime }\right)$, and $y^{\prime }∼p_{\theta_{t}}\left(\cdot |x^{\prime }\right)$.

Subsequently, we use an executor to filter the responses generated by the adversary model in each round on the database:

(11) $$\left(y_{\mathrm{right}}^{\prime },y_{\mathrm{wrong}}^{\prime }\right)=\mathrm{Execute}\left(y^{\prime },\mathrm{Database}\right)$$

By discarding the correct SQL statements $y_{\mathrm{right}}^{\prime }$ generated by the adversary model, we retain only the incorrect SQL statements $y_{\mathrm{wrong}}^{\prime }$ and use them to update the objective function of the main model:

(12) $$f_{t+1}=\underset{f\in F_{t}}{\mathrm{argmin}}E\left[\ell \left(f\left(x^{\prime },y\right)-f\left(x^{\prime },y_{\mathrm{wrong}}^{\prime }\right)\right)\right]$$

For a given $f_{t+1}$ and the response *y* to $x^{\prime }$, the value $f_{t+1}\left(x^{\prime },y\right)$ reflects the main model’s ability to distinguish the outputs of the adversary model. Ideally, when $y∼p_{\mathrm{data}}\left(\cdot |x^{\prime }\right)$, $f_{t+1}$ should output a higher value, whereas, when $y_{\mathrm{wrong}}^{\prime }∼p_{\theta_{t}}\left(\cdot |x^{\prime }\right)$, it should output a lower value. After obtaining the discrimination results from the main model, we update the parameters $\theta_{t+1}$ of the adversary model. The objective function for updating the adversary model is as follows:

(13) $$E_{x^{\prime }∼q\left(\cdot \right),y∼p\left(\cdot |x^{\prime }\right)}\left[f_{t+1}\left(x^{\prime },y_{\mathrm{wrong}}^{\prime }\right)\right]$$

Finally, we integrate the two aforementioned steps into an end-to-end training objective and derive our final loss function:

(14) $$L_{\mathrm{ExSPIN}}=E\left[\ell \left(\lambda \log\frac{p_{\theta }\left(y|x^{\prime }\right)}{p_{\theta_{t}}\left(y|x^{\prime }\right)}-\lambda \log\frac{p_{\theta }\left(y_{\mathrm{wrong}}^{\prime }|x^{\prime }\right)}{p_{\theta_{t}}\left(y_{\mathrm{wrong}}^{\prime }|x^{\prime }\right)}\right)\right]$$

This formula represents the ultimate training objective of ExSPIN. Unlike SPIN, we have filtered out training samples where the adversary model generates incorrect SQL $y_{\mathrm{wrong}}^{\prime }$ through the execution feedback mechanism. By calculating the difference in the log probability ratios between the correctly human-annotated SQL *y* and the incorrect SQL $y_{\mathrm{wrong}}^{\prime }$, we guide the main model to adjust its outputs to more closely align with *y*, thereby correcting the errors of the adversary model.

We then iteratively repeat this self-play process, allowing the model to progressively deepen its understanding of the database schema links, ultimately generating high-quality SQL queries.

Our theoretical framework is analogous to adversarial games, where the model continuously improves through competition with its past versions. Specifically, in ExSPIN, under the execution feedback mechanism, the model applies a penalty to incorrect SQL queries generated by its opponent model:

(15) $$P=-\lambda \log p_{\theta }\left(y_{\mathrm{wrong}}^{\prime }|x^{\prime }\right)$$

This penalty term guides the model to identify and correct errors in the opponent model’s generated SQL queries, helping it to recognize its own weaknesses and produce more accurate and executable SQL queries. At the same time, the model applies a reward to correctly annotated SQL queries:

(16) $$R=\lambda \log p_{\theta }\left(y|x\right)$$

This reward term encourages the model to modify its outputs to better align with correct SQL queries, effectively guiding the model to update itself in the right direction. Through this iterative self-play process, the model progressively enhances its text-to-SQL performance.

Using the executor, we filter out correct and incorrect SQL statements generated by the adversary model. To maintain model robustness, we discard correctly executed results and use only incorrect results for SPIN. This approach ensures that the main player model negates only incorrect SQL statements while acknowledging correct ones. As a result, the SQL generation accuracy improves during self-play. The detailed algorithm is presented in Algorithm 2, Given a query $x_{i}\in Q$ and an initial model $p_{\theta_{0}}$, we first use explicit schema integration to obtain $Prompt_{\mathrm{db}}$. By concatenating $Prompt_{\mathrm{db}}$ with $x_{i}\in Q$, we construct the model input $x_{i}^{\prime }$. The model is then divided into a main player model and an opponent model. The opponent model generates SQL queries, and an executor is used to filter out incorrect SQL queries. These filtered data are used to train the main player model, resulting in the next iteration of the model. Finally, the opponent model is updated to the newly trained model. In this process, the opponent model for each round is the result of the training from the previous round.   
**Algorithm 2:** ExSPIN
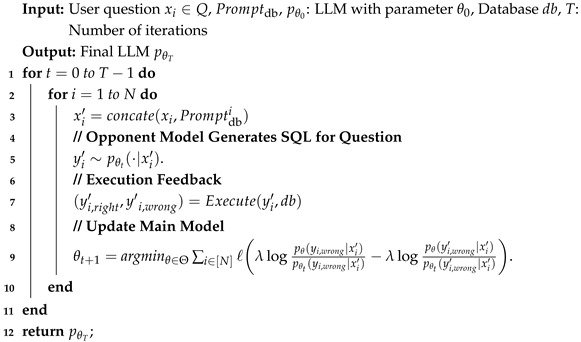


## 5. Experiments

### 5.1. Experimental Setup

#### 5.1.1. Datasets

We perform testing on the following two real-world benchmarks.

**SPIDER.** The dataset [16] is divided into a training set (8659 samples), a development set (1034 samples), and a test set. Of the 7000 samples in the training set, 1659 are sourced from six previously released text-to-SQL datasets, including Restaurants, GeoQuery, Scholar, Academic, IMDB, and Yelp. The SPIDER dataset comprises 200 databases spanning 138 different domains. We evaluate our method using the test set provided by the SPIDER dataset, which includes 2147 test queries and 40 test databases.

**BIRD.** The dataset [34] consists of 12,751 question–SQL pairs across 37 domains, including finance, healthcare, education, and more. These queries are carefully crafted to reflect the complexities of real-world scenarios, such as handling large databases (some exceeding 10,000 rows), implicit domain knowledge, and noisy or ambiguous natural language queries. Additionally, BIRD prioritizes execution accuracy over syntactic correctness, ensuring that models are evaluated not only on their ability to generate valid SQL but also on their capacity to produce queries that yield correct and meaningful results when executed on real databases.

#### 5.1.2. Models

We evaluated our approach across four distinct open-source LLMs for code generation, with parameter sizes ranging from 1.5B to 14B.

**DeepSeek-Coder-Instruct 6.7B:** The DeepSeek-Coder [6] series is a comprehensive suite of code LLMs, each meticulously trained from the ground up on an extensive dataset comprising 2 trillion tokens. This dataset consists of 87% code and 13% natural language, spanning both English and Chinese. The series includes models of varying sizes, ranging from 6.7 billion to 33 billion parameters, designed to support a wide range of application scenarios. All models are pre-trained on a project-level code corpus, utilizing a 16,000-token context window and incorporating a fill-in-the-blank task to enhance their proficiency in project-level code completion and infilling. In terms of coding capabilities, DeepSeek-Coder sets new benchmarks among open-source code models across multiple programming languages and evaluation benchmarks, demonstrating its exceptional proficiency in code comprehension and generation.

**Qwen2.5-Coder-Instruct 1.5B/7B/14B:** Qwen2.5-Coder [35] represents the evolution of open-source coding models, succeeding CodeQwen1.5. Available in 1.5 billion, 7 billion, 14 billion, and 32 billion parameter versions, it is trained on an enormous 5.5 trillion tokens, which include code, text-code grounding, and synthetic data. This extensive training regimen significantly enhances its coding capabilities while preserving its strong performance in mathematical and general tasks. Supporting up to 128,000 tokens and 92 programming languages, Qwen2.5-Coder excels in code generation, completion, and repair. Its instruction-tuned variant, Qwen2.5-Coder-Instruct, further refines task performance and generalization, demonstrating exceptional skill in multi-programming, code reasoning, and mathematical tasks, while maintaining robust general capabilities.

#### 5.1.3. Baselines

To evaluate the performance of our ExSPIN, we use two SOTA fine-tuning methods, SPIN [14] and SFT [36], as baselines.

#### 5.1.4. Metrics

Following [2], we evaluate the performance of our text-to-SQL model using the execution accuracy (EX) as the primary metric. This metric measures whether the SQL query generated by the model, when executed on the database, produces the same result as the execution of the ground truth (gold) SQL query.

### 5.2. Overall Comparison

**Figure 4:**
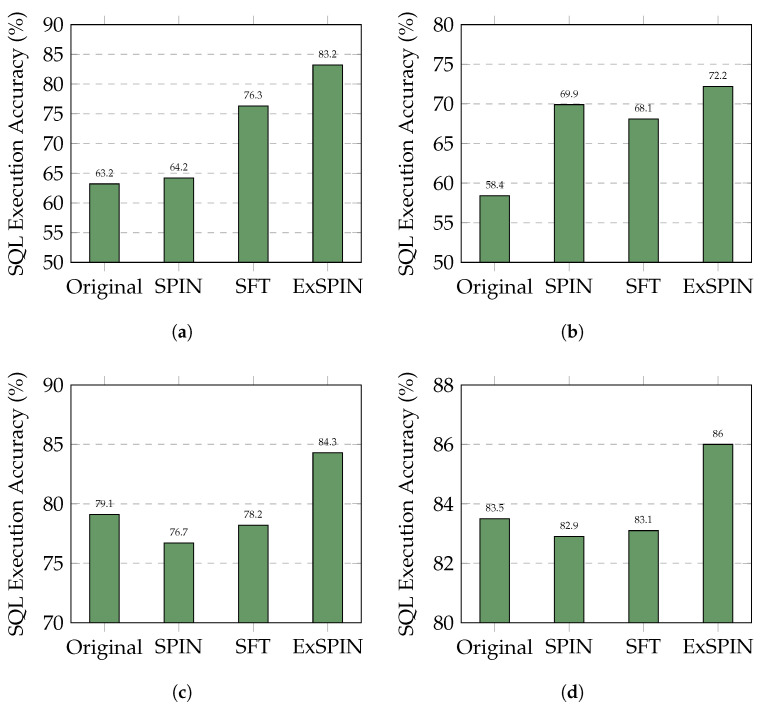
Comparison results of different LLMs with different methods on the SPIDER dataset. The original represents the original LLM, while SFT, SPIN, and ExSPIN denote the models fine-tuned on the training set using SFT, SPIN, and ExSPIN, respectively. (**a**) DeepSeek-Coder-Instruct 6.7B. (**b**) Qwen2.5-Coder-Instruct 1.5B. (**c**) Qwen2.5-Coder-Instruct 7B. (**d**) Qwen2.5-Coder-Instruct 14B.

**Figure 5:**
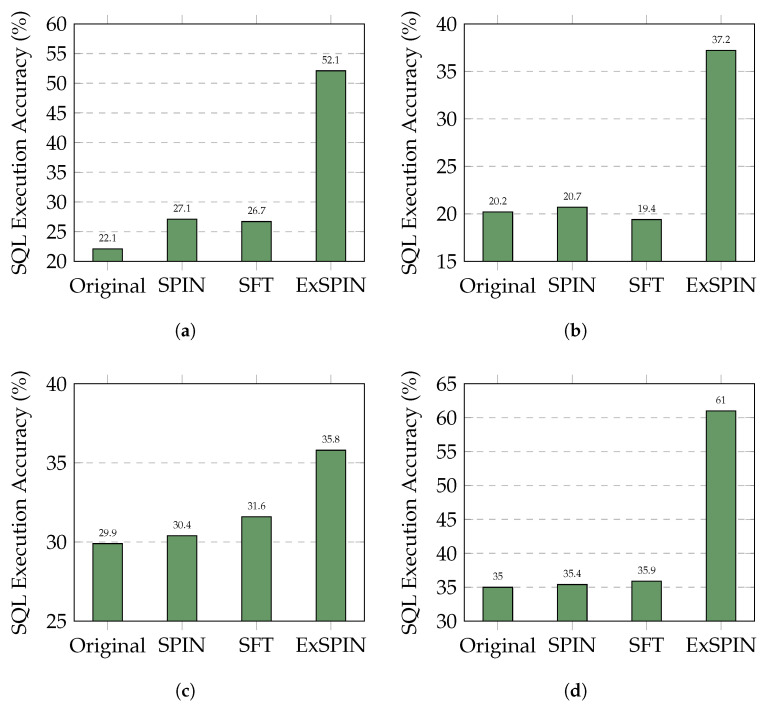
Comparison results of different LLMs with different methods on the BIRD dataset. The original represents the original LLM, while SFT, SPIN, and ExSPIN denote the models fine-tuned on the training set using SFT, SPIN, and ExSPIN, respectively. (**a**) DeepSeek-Coder-Instruct 6.7B. (**b**) Qwen2.5-Coder-Instruct 1.5B. (**c**) Qwen2.5-Coder-Instruct 7B. (**d**) Qwen2.5-Coder-Instruct 14B.

Figure 4 and Figure 5 show the execution accuracy of the SQL queries generated on the SPIDER and BIRD datasets when applying SFT, SPIN, and ExSPIN to four different models. For both SPIN and ExSPIN, we report the best results across four iterations. The experimental results reveal that the SPIN method did not positively impact the SFT models. On the SPIDER dataset, SPIN achieved accuracy of only 64.2% on the DeepSeek-Coder 6.7B model, representing a 12% decrease compared to SFT. On the BIRD dataset, SPIN reached accuracy of just 30.4% on the Qwen2.5-Coder 7B model, a reduction of 1% compared to SFT. These findings suggest that SPIN failed to improve the models through self-play on top of SFT, instead leading to incorrect updates and degraded performance. This failure can be attributed to the SPIN training process, where the main player model is trained to distinguish between SQL queries generated by the opponent model across the entire dataset and adjust its parameters to avoid generating queries similar to the opponent’s. However, in the text-to-SQL task, the opponent model may generate correct SQL queries. As a result, SPIN inadvertently guides the main player model toward producing incorrect answers. This issue is less pronounced when the opponent model generates SQL queries with low accuracy, such as with the Qwen2.5-Coder 1.5B model.

In nearly all cases, ExSPIN achieves superior performance. For example, on the SPIDER dataset with the DeepSeek-Coder 6.7B model, ExSPIN reached accuracy of 83.2%, representing a 19.0% improvement over SPIN. Similarly, on the BIRD dataset, ExSPIN achieved accuracy of 52.1%, a 25.0% increase compared to SPIN. This performance boost can be attributed to the integration of the execution feedback mechanism, which addresses SPIN’s shortcomings, while explicit schema integration enhances the model’s understanding of the relationships between the query and the database. By embedding schema information into the self-play fine-tuning process through techniques such as schema filtering, value retrieval, and metadata integration, ExSPIN overcomes the inherent limitations of traditional SPIN. The inclusion of schema-linking allows the model to more effectively align natural language queries with the underlying database structure, especially in complex scenarios involving multi-table joins, precise value matching, and a deeper understanding of schema relationships. This capability significantly reduces the semantic gap between natural language and SQL, resulting in more accurate queries.

Table 1 and Table 2 show the accuracy of different types of queries on the SPIDER and BIRD datasets using Qwen2.5-Coder 14B, respectively. In almost all cases, our ExSPIN model achieves the best performance. The experimental results indicate that both the SFT and SPIN methods struggle with three specific types of SQL queries: join queries without aggregates, aggregate queries with join and group by, and nested subqueries. On the SPIDER dataset, the accuracy of both the SFT and SPIN methods for these query types did not exceed 80%, with SPIN achieving only 73.1% accuracy on nested subqueries. On the BIRD dataset, the accuracy for nested subqueries dropped even further, with SFT and SPIN achieving only 28% and 27.7%, respectively. This performance gap arises because the handling of join operations and nested subqueries requires a clear understanding of the relationships between the database tables. However, the SPIN method relies solely on implicit associations between natural language queries and the database schema, which limits the model’s ability to accurately capture the underlying data relationships. In contrast, ExSPIN explicitly integrates schema and execution feedback information, providing the model with direct access to database structural features. This enhancement significantly improves the model’s ability to capture relationships between tables.

Finally, we conducted comparative experiments involving GPT-4o and the DPO [24] method, where SFT, SPIN, DPO, and ExSPIN were implemented on the DeepSeek-Coder 6.7B model. The experimental results are shown in Table 3. The experimental findings indicate that, while the DPO method still occasionally misguides the model into altering its correct outputs, its impact on the model’s accuracy is relatively minor compared to the SPIN method, due to the smaller number of parameters involved in fine-tuning. The ExSPIN method continues to demonstrate significant advantages over DPO, achieving improvements of 15.9% on the SPIDER dataset and 24.3% on the BIRD dataset. Compared to GPT-4o, by fine-tuning the DeepSeek 6.7B model, ExSPIN achieved a 7.1% lead on the SPIDER dataset and a 6% lead on the BIRD dataset. Since some of the databases and question–answer pairs in the SPIDER test set differ from those in the training set, they can be considered as unseen data for the model. The experimental results show that ExSPIN outperforms other methods on the SPIDER test set, demonstrating the generalization ability of our approach to unseen databases.

### 5.3. Parameter Study

**Figure 6:**
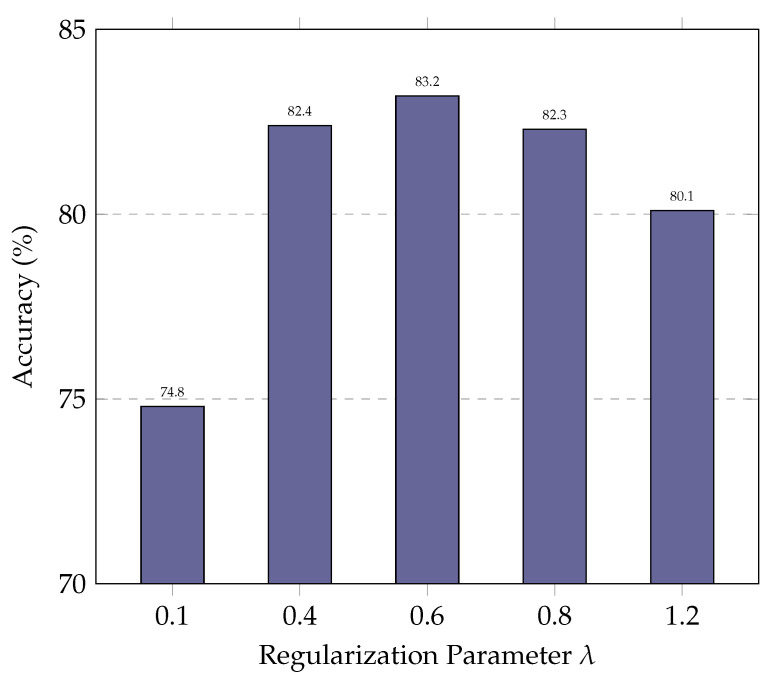
Performance of ExSPIN with various λ parameters on the SPIDER dataset.

**Figure 7:**
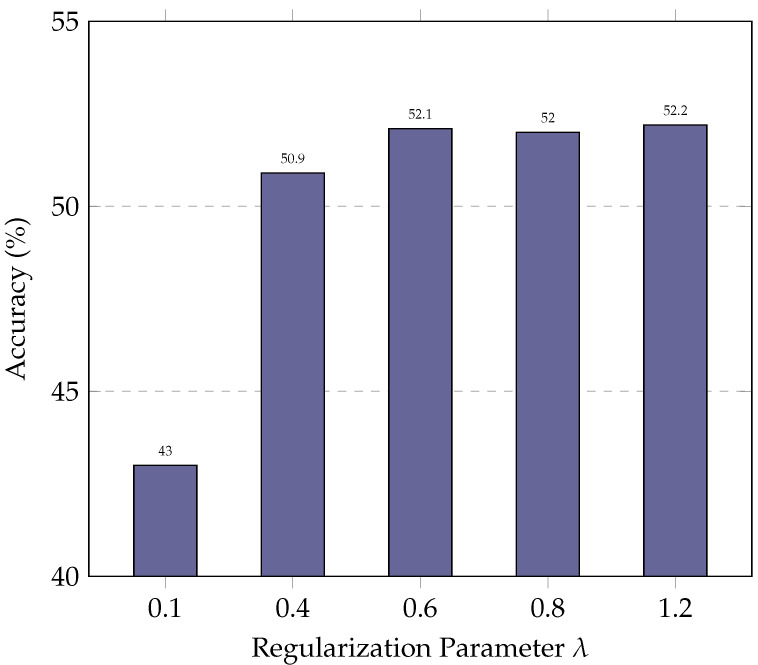
Performance of ExSPIN with various λ parameters on the BIRD dataset.

In this subsection, we evaluate the impact of the regularization parameter λ on ExSPIN training with the DeepSeek-Coder 6.7B. The values of λ are varied between 0.1 and 1.2. As shown in Figure 6 and Figure 7, the experimental results indicate that a low λ limits the model’s ability to update its parameters effectively. As a result, the model fails to leverage errors in the generated SQL during training, leading to only marginal improvements in accuracy. On the other hand, an excessively high λ leads to the over-penalization of both the log probabilities of the gold SQL and the generated SQL, with the model attempting to maximize the gap between their log probabilities. This, in turn, reduces the similarity between the generated SQL and the gold SQL, making it more difficult for the model to produce high-quality queries.

### 5.4. Execution Feedback Mechanism Analysis

To better illustrate the improvement of the execution feedback mechanism in self-play fine-tuning, we have selected two examples to demonstrate the impact of execution feedback on SQL generation.

Table 4 presents the first example, where we observe that the model successfully generates the correct SQL under the guidance of explicit schema integration after SFT (Section 4.2). We then conducted self-play experiments both with and without the execution feedback mechanism. As shown in Table 4, the SQL generated without execution feedback is incorrect, whereas correct results are generated when execution feedback is included. This example illustrates that, while the SFT model can generate correct SQL statements under explicit schema integration, there is a notable difference between the generated SQL and the gold SQL in terms of grammatical structure. In the absence of the execution feedback mechanism, the model alters its originally correct output during self-play, ultimately failing to generate the correct SQL. Furthermore, this process reduces the diversity of the SQL outputs and weakens the model’s generalization ability. In contrast, when the feedback mechanism is introduced, misleading samples are filtered out during training. This prevents the model from being confused by its own outputs and ensures that it consistently generates correct SQL statements.

Table 5 shows the second example, where the model still fails to generate the correct SQL even after SFT. Consequently, this sample is retained for self-play, where the model corrects the erroneous outputs of its opponent model and learns from the gold SQL. As a result, the model eventually produces the correct SQL, thereby improving its ability to handle the text-to-SQL task.

These two cases demonstrate that the execution feedback mechanism plays a crucial role in self-play. It enables the model to correctly revise its mistakes while retaining the correct SQL outputs that it has already learned, ultimately improving its overall performance on text-to-SQL tasks.

### 5.5. Ablation Study

Finally, we conducted ablation experiments on ExSPIN with DeepSeek-Coder 6.7B to answer the following two questions:

1. How does explicitly incorporating database features into the model affect its ability to understand the relationship between the database and the query?
2. What is the impact of introducing an execution feedback mechanism during the SPIN process on the model’s SQL generation capabilities in self-play?

Table 6 and Table 7 present the results of our ablation experiments on the two datasets. The experimental results show that explicit schema integration contributes an 8% improvement in the SQL generation accuracy, as it helps the model to better identify relevant tables, columns, and query structures from the database in relation to the input question. Additionally, we observe that the execution feedback mechanism plays a crucial role in guiding multi-round self-play training, enabling the model to correct its own erroneous SQL queries and avoid modifying correct SQL statements. As a result, the SQL generation accuracy was improved from 81.6% to 83.2% on SPIDER and 39.6% to 52.1% on BIRD.

### 5.6. Resource Consumption

Table 8 and Table 9 presents a comparison of the resource consumption during the training processes of SPIN, ExSPIN, and SFT on the SPIDER and BIRD datasets. The experimental results demonstrate that ExSPIN, when fine-tuning the DeepSeek-Coder-6.7B-Instruct model on the SPIDER dataset, utilizes four A800 GPUs and consumes 51.25 min per epoch. In contrast, the SPIN method requires 203.5 min per epoch. This significant difference arises because ExSPIN employs an execution feedback mechanism to filter out correct examples that the current model can already generate before training, retaining only a small subset of incorrect examples for fine-tuning. On the other hand, SPIN trains on the entire dataset in each iteration, resulting in substantially higher time consumption compared to ExSPIN. Additionally, we observe that ExSPIN consumes only 31.27 min per epoch when fine-tuning the Qwen2.5-Coder-14B model. This is attributed to the stronger baseline capabilities of Qwen2.5-Coder-14B compared to DeepSeek-Coder-6.7B-Instruct, which lead to fewer remaining bad cases after the execution feedback mechanism filters the dataset, thereby reducing the training time. This suggests that our approach is more scalable to larger and more complex datasets compared to SPIN.

### 5.7. Bad Case Analysis

The performance on different SQL query types in Section 5.2 reveals that ExSPIN still has limitations in handling nested subqueries, as overly complex SQL query patterns remain challenging for the current model. Table 10 presents examples of the errors encountered by ExSPIN when processing nested subqueries. These examples demonstrate that when SQL statements contain structurally complex nested subqueries, ExSPIN struggles to generate properly structured subqueries, often producing oversimplified subqueries or failing to generate them altogether. Additionally, the model faces difficulties in managing UNION relationships between subqueries. These findings indicate that ExSPIN’s capability in handling complex nested subqueries requires further improvement. Nevertheless, our method has achieved a significant improvement in accuracy for nested subqueries, outperforming SFT and SPIN by 5.3% to 14.2%.

### 5.8. Limitations

**Figure 8:**
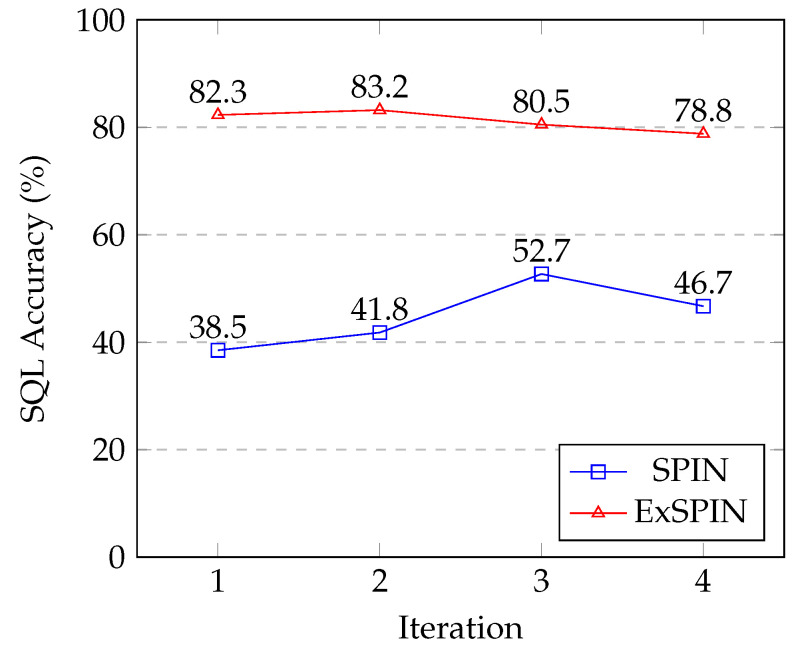
The multi-round iteration results of SPIN and ExSPIN on the SPIDER dataset.

**Figure 9:**
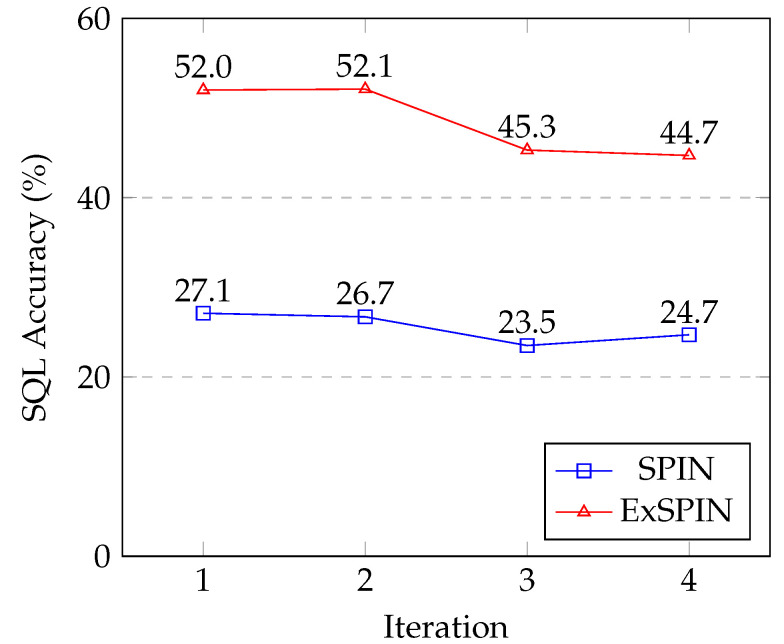
The multi-round iteration results of SPIN and ExSPIN on the BIRD dataset.

Figure 8 and Figure 9 show the SQL execution accuracy at each iteration for SPIN and ExSPIN across multiple rounds of training. The experimental results reveal a downward trend in the SQL execution accuracy as the number of training iterations increases. This decline is attributed to the fact that both SPIN and ExSPIN perform iterative training on the same dataset, which increases the likelihood of overfitting to the training set after several iterations. In future work, we plan to investigate methods such as dataset partitioning to mitigate the risk of overfitting during iterative training.

Secondly, regarding the impact of database biases on model performance, since our method assumes that the distributions of the training and test sets are the same, during the multi-turn self-play fine-tuning, the model learns the distribution of the databases and SQL in the training set. If there are biases in the dataset, this will inherently limit the potential of the current self-play approach. Surprisingly, the experiments shown in Table 3 of Section 5.2 on the SPIDER test set indicate that our method has potential resistance to dataset bias. Despite the existence of schema and SQL-type biases between the SPIDER test set and training set, our method still improves the performance by 6.9% to 19% compared to the SFT, SPIN, GPT-4o, and DPO methods on the SPIDER test set.

Finally, compared to SFT, the ExSPIN method consumes more memory and requires longer training times, which is indeed a limitation of our approach. We plan to explore ways to reduce the computational overhead in the future.

## 6. Conclusions

In this work, we have introduced ExSPIN, a novel explicit feedback-based self-play fine-tuning framework designed to address the limitations of conventional SPIN methods in text-to-SQL parsing. By incorporating schema-linking features and SQL execution results explicitly into the training process, our method bridges the gap between natural language queries and database schemas, enabling the model to generate more accurate SQL queries. Through schema filtering, value retrieval, and metadata integration, ExSPIN demonstrates a significant improvement over existing methods, as evidenced by its superior performance on the SPIDER dataset. These results highlight the importance of leveraging explicit schema-linking in self-play fine-tuning, paving the way for more robust and efficient text-to-SQL parsers. We believe that our approach sets a strong foundation for future research in combining schema-aware techniques with large language models and offers promising opportunities for enhanced database interaction systems. 

## Figures and Tables

**Table 1:** Examples of different SQL query types on the SPIDER dataset.

Method	Equality Select by Key	Equality Select by Non-Key	Join Queries Without Aggregates	Aggregate Queries with Group by	Aggregate with Join and Group by	Nested Subqueries	Total
SFT	92.5	90.4	76.3	87.5	78.2	73.4	83.1
SPIN	**92.5**	90.8	74.7	87.8	78.0	73.1	82.9
ExSPIN	90.3	**92.2**	**81.2**	**89.5**	**81.8**	**78.7**	**86.0**

**Table 2:** Examples of different SQL query types on the BIRD dataset.

Method	Equality Select by Key	Equality Select by Non-Key	Join Queries Without Aggregates	Aggregate Queries with Group by	Aggregate with Join and Group by	Nested Subqueries	Total
SFT	44.0	42.2	40.7	31.8	28.7	28.0	35.9
SPIN	44.0	42.5	38.2	32.1	28.4	27.7	35.4
ExSPIN	**66.0**	**63.9**	**62.1**	**68.8**	**59.0**	**41.9**	**61.0**

**Table 3:** Comparison of different methods.

Method	SPIDER	BIRD
SFT	76.3	26.7
SPIN	64.2	27.1
GPT-4o	76.1	46.1
DPO	67.3	27.8
ExSPIN	**83.2**	**52.1**

**Table 4:** The impact of execution feedback on SFT’s right case.

Stage	Details
**Before self-play fine-tuning**	**Question**: How many accounts who have a region in Prague are eligible for loans?
	**Pred SQL**: SELECT count(loan.account_id) FROM district INNER JOIN account ON district.district_id = account.district_id INNER JOIN loan ON account.account_id = loan.account_id WHERE district.a3 = ’Prague’
	**Gold SQL**: SELECT COUNT(T1.account_id) FROM account AS T1 INNER JOIN loan AS T2 ON T1.account_id = T2.account_id INNER JOIN district AS T3 ON T1.district_id = T3.district_id WHERE T3.A3 = ’Prague’
	**Result**: right
**ExSPIN without feedback**	**Question**: How many accounts who have region in Prague are eligible for loans?
	**Pred SQL**: SELECT COUNT(T1.account_id) FROM account AS T1 INNER JOIN district AS T2 ON T1.district_id = T2.district_id WHERE T2.A14 = 1
	**Gold SQL**: SELECT COUNT(T1.account_id) FROM account AS T1 INNER JOIN loan AS T2 ON T1.account_id = T2.account_id INNER JOIN district AS T3 ON T1.district_id = T3.district_id WHERE T3.A3 = ’Prague’
	**Result**: wrong
**ExSPIN**	**Question**: How many accounts who have region in Prague are eligible for loans?
	**Pred SQL**: SELECT count(loan.account_id) FROM district INNER JOIN account ON district.district_id = account.district_id INNER JOIN loan ON account.account_id = loan.account_id WHERE district.a3 = ’Prague’
	**Gold SQL**: SELECT COUNT(T1.account_id) FROM account AS T1 INNER JOIN loan AS T2 ON T1.account_id = T2.account_id INNER JOIN district AS T3 ON T1.district_id = T3.district_id WHERE T3.A3 = ’Prague’
	**Result**: right

**Table 5:** The impact of execution feedback on SFT’s bad case.

Stage	Details
**Before self-play fine-tuning**	**Question**: Among the schools with an average score in Math over 560 in the SAT test, how many schools are directly charter-funded?
	**Pred SQL**: SELECT COUNT(*) FROM schools AS T1 INNER JOIN satscores AS T2 ON T1.CDSCode = T2.cds WHERE T2.AvgScrMath > 560 AND T1.StatusType = ‘Directly Chartered’
	**Gold SQL**: SELECT COUNT(T2.‘School Code’) FROM satscores AS T1 INNER JOIN frpm AS T2 ON T1.cds = T2.CDSCode WHERE T1.AvgScrMath > 560 AND T2.‘Charter Funding Type’ = ‘Directly funded’
	**Result**: wrong
**ExSPIN without feedback**	**Question**: Among the schools with an average score in Math over 560 in the SAT test, how many schools are directly charter-funded?
	**Pred SQL**: SELECT count(schools.cdscode) FROM schools INNER JOIN satscores ON schools.cdscode = satscores.cds WHERE satscores.avgscrmath > 560 AND schools.fundingtype = ‘Directly funded’
	**Gold SQL**: SELECT COUNT(T2.‘School Code’) FROM satscores AS T1 INNER JOIN frpm AS T2 ON T1.cds = T2.CDSCode WHERE T1.AvgScrMath > 560 AND T2.‘Charter Funding Type’ = ‘Directly funded’
	**Result**: right
**ExSPIN**	**Question**: Among the schools with an average score in Math over 560 in the SAT test, how many schools are directly charter-funded?
	**Pred SQL**: SELECT count(schools.cdscode) FROM satscores INNER JOIN schools ON satscores.cds = schools.cdscode WHERE satscores.avgscrmath > 560 AND schools.fundingtype = ‘Directly funded’
	**Gold SQL**: SELECT COUNT(T2.‘School Code’) FROM satscores AS T1 INNER JOIN frpm AS T2 ON T1.cds = T2.CDSCode WHERE T1.AvgScrMath > 560 AND T2.‘Charter Funding Type’ = ‘Directly funded’
	**Result**: right

**Table 6:** Ablation study of ExSPIN on the SPIDER dataset.

Ablation	iter0	iter1	iter2	iter3
Overall	82.3	83.2	80.5	78.8
w/o Explicit Schema Integration	73.3	75.3	72.2	69.6
w/o Execute Feedback	81.6	77.9	75.1	71.4

**Table 7:** Ablation study of ExSPIN on the BIRD dataset.

Ablation	iter0	iter1	iter2	iter3
Overall	52.0	52.1	45.3	44.7
w/o Explicit Schema Integration	28.6	28.7	27.6	26.9
w/o Execute Feedback	39.6	31.9	29.5	28.5

**Table 8:** Resource consumption on SPIDER.

Model	Method	GPUs	Time/Epoch (min)	Memory (GB)
DeepSeek-Coder-6.7B	ExSPIN	4 × A800	51.25	278
	SPIN	4 × A800	203.5	297
	SFT	4 × A800	17.5	51
Qwen2.5-Coder-14B	ExSPIN	8 × A800	31.27	627
	SPIN	8 × A800	412	635
	SFT	4 × A800	22	115

**Table 9:** Resource consumption on BIRD.

Model	Method	GPUs	Time/Epoch (min)	Memory (GB)
DeepSeek-Coder-6.7B	ExSPIN	4 × A800	84.3	296
	SPIN	4 × A800	242	314
	SFT	4 × A800	19.27	59
Qwen2.5-Coder-14B	ExSPIN	8 × A800	107.5	629
	SPIN	8 × A800	489	632
	SFT	4 × A800	48.6	115

**Table 10:** ExSPIN’s bad case examples.

Details
**Gold SQL**: SELECT ‘date’ FROM (SELECT t2.crossing, t2.‘date’ FROM Player AS t1 INNER JOIN Player_Attributes AS t2 ON t1.player_fifa_api_id = t2.player_fifa_api_id WHERE t1.player_name = ’Kevin Constant’ ORDER BY t2.crossing DESC) ORDER BY date DESC LIMIT 1
**Pred SQL**: SELECT T1.date FROM Player_Attributes AS T1 INNER JOIN Player AS T2 ON T1.player_api_id = T2.player_api_id WHERE T2.player_name = ‘Kevin Constant’ ORDER BY T1.crossing DESC LIMIT 1
**Result**: wrong
**Gold SQL**: SELECT A FROM (SELECT AVG(finishing) result, ‘Max’ A FROM Player AS T1 INNER JOIN Player_Attributes AS T2 ON T1.player_api_id = T2.player_api_id WHERE T1.height = (SELECT MAX(height) FROM Player) UNION SELECT AVG(finishing) result, ‘Min’ A FROM Player AS T1 INNER JOIN Player_Attributes AS T2 ON T1.player_api_id = T2.player_api_id WHERE T1.height = (SELECT MIN(height) FROM Player)) ORDER BY result DESC LIMIT 1
**Pred SQL**: SELECT T1.player_name FROM Player AS T1 INNER JOIN Player_Attributes AS T2 ON T1.player_api_id = T2.player_api_id WHERE T1.height = (SELECT MAX(height) FROM Player) OR T1.height = (SELECT MIN(height) FROM Player) ORDER BY T2.finishing DESC LIMIT 1
**Result**: wrong

## Data Availability

The original contributions presented in this study are included in the article. Further inquiries can be directed to the corresponding authors.
